# Supplementary material for: Long-term potentiation prevents ketamine-induced aberrant neurophysiological dynamics in the hippocampus-prefrontal cortex pathway in vivo
Source: Sci Rep. 2020 Apr 28;10:7167. doi: 10.1038/s41598-020-63979-5 (PMC7188848; doi:10.1038/s41598-020-63979-5)
Supplement: Supplementary file 1 — Supplementary information. [file 41598_2020_63979_MOESM1_ESM.pdf]

Long-term potentiation prevents ketamine-induced aberrant neurophysiological dynamics in the hippocampus-prefrontal cortex pathway *in vivo*

Cleiton Lopes-Aguiar[1+], Rafael N. Ruggiero[2+*], Matheus T. Rossignoli[2], Ingrid de Miranda Esteves[2], José Eduardo Peixoto-Santos[3], Rodrigo N. Romcy-Pereira[4], João P. Leite[2].

[1]Núcleo de Neurociências, Department of Physiology and Biophysics, Institute of Biological Sciences, Federal University of Minas Gerais, Belo Horizonte, 31270-901, Brazil

[2]Department of Neuroscience and Behavioral Sciences, Ribeirão Preto Medical School, University of São Paulo, Ribeirão Preto, 14049-900, Brazil

[3]Department of Neurology and Neurosurgery, UNIFESP, Sao Paulo, SP, 04039-032, Brazil

[4]Brain Institute, Federal University of Rio Grande do Norte, Natal, RN 59056-450, Brazil

*rafaruggiero@usp.br

+these authors contributed equally to this work

Supplementary figures

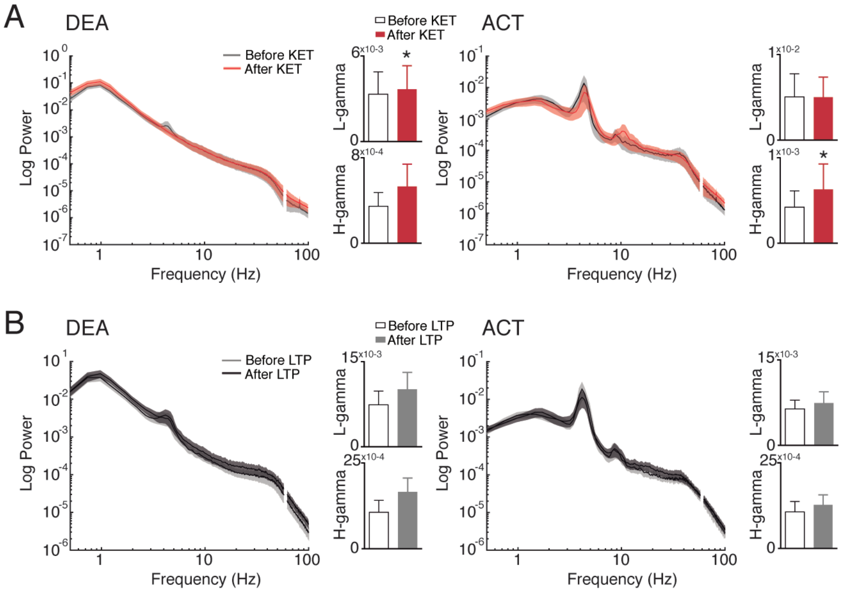

Figure S1 – KET and HFS effects on HPC power spectral density. (A) Ketamine produced a subtle low-gamma increase in DEA (left, Wilcoxon test, n=7, p=0.0469) and high-gamma (right, Wilcoxon test, n=7, p=0.0312) in ACT states (right). (B) LTP induction did not produce a gamma increase in DEA (left) or ACT states (right). Line plots representing average PSDs in the HPC before and after ketamine injection. Bar plots representing low and high-gamma frequency bands before and after ketamine administration. *(p<0.05).

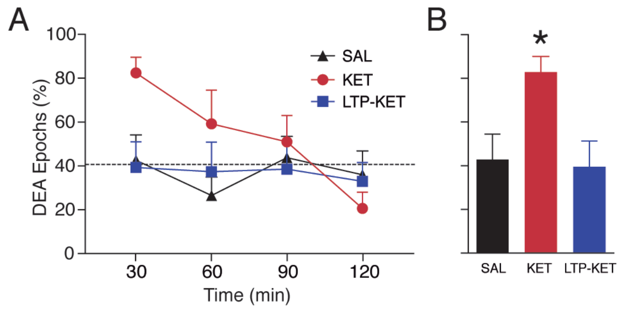

Figure S2 – Ketamine administration effects on the brain state alternation under urethane anesthesia. (A) Time-course of the average percentage of DEA epochs percentage after KET or Sal administration (left). The dashed line represents the chance of obtaining a DEA epoch in a 30 min period calculated by permuting all epochs from all groups 40,000 times. (B) Average percentage of DEA epochs in the initial 30 min after drug administration. Ketamine produces an acute (30 min) increase in brain states epochs classified as DEA. One-way ANOVA: $F_{(2,21)}=4.64$, $p=0.0214$, Tukey post-hoc test $p=0.049$ for KET vs. Sal, and $p=0.0271$ for KET vs. LTP-KET. Interestingly, LTP induction prevented KET increase in DEA epochs. *($p<0.05$).
